# Natural killer cells attenuate cytomegalovirus-induced hearing loss in mice

**DOI:** 10.1371/journal.ppat.1006599

**Published:** 2017-08-31

**Authors:** Ali A. Almishaal, Pranav D. Mathur, Elaine Hillas, Liting Chen, Anne Zhang, Jun Yang, Yong Wang, Wayne M. Yokoyama, Matthew A. Firpo, Albert H. Park

**Affiliations:** 1 Department of Communication Sciences and Disorders, University of Utah College of Health, Salt Lake City, Utah, United States of America; 2 Department of Ophthalmology and Visual Sciences, University of Utah School of Medicine, Salt Lake City, Utah, United States of America; 3 Department of Neurobiology and Anatomy, University of Utah School of Medicine, Salt Lake City, Utah, United States of America; 4 Department of Surgery, University of Utah School of Medicine, Salt Lake City, Utah, United States of America; 5 Division of Otolaryngology, University of Utah School of Medicine, Salt Lake City, Utah, United States of America; 6 Division of Rheumatology, Washington University School of Medicine, St. Louis, Missouri, United States of America; 7 Howard Hughes Medical Institute, Washington University School of Medicine, St. Louis, Missouri, United States of America; University of Wisconsin-Madison, UNITED STATES

## Abstract

Congenital cytomegalovirus (CMV) infection is the most common non-hereditary cause of sensorineural hearing loss (SNHL) yet the mechanisms of hearing loss remain obscure. Natural Killer (NK) cells play a critical role in regulating murine CMV infection via NK cell recognition of the Ly49H cell surface receptor of the viral-encoded m157 ligand expressed at the infected cell surface. This Ly49H NK receptor/m157 ligand interaction has been found to mediate host resistance to CMV in the spleen, and lung, but is much less effective in the liver, so it is not known if this interaction is important in the context of SNHL. Using a murine model for CMV-induced labyrinthitis, we have demonstrated that the Ly49H/m157 interaction mediates host resistance in the temporal bone. BALB/c mice, which lack functional Ly49H, inoculated with mCMV at post-natal day 3 developed profound hearing loss and significant outer hair cell loss by 28 days of life. In contrast, C57BL/6 mice, competent for the Ly49H/m157 interaction, had minimal hearing loss and attenuated outer hair cell loss with the same mCMV dose. Administration of Ly49H blocking antibody or inoculation with a mCMV viral strain deleted for the m157 gene rendered the previously resistant C57BL/6 mouse strain susceptible to hearing loss to a similar extent as the BALB/c mouse strain indicating a direct role of the Ly49H/m157 interaction in mCMV-dependent hearing loss. Additionally, NK cell recruitment to sites of infection was evident in the temporal bone of inoculated susceptible mouse strains. These results demonstrate participation of NK cells in protection from CMV-induced labyrinthitis and SNHL in mice.

## Author summary

Cytomegalovirus (CMV) transmission from an infected mother to her fetus is a leading cause of permanent hearing loss in children, but the contributing processes are not clear. In this report, we utilized a mouse model, which recapitulates many features of congenital CMV mediated childhood hearing loss, to demonstrate that natural killer cells (NK), a component of early host immune response to infection, play a critical protective role in CMV-induced hearing loss. Specifically, we determined that NK cells interact with CMV infected cells through binding of the NK cell receptor, Ly49H, with a virally-encoded protein, m157, expressed on the cell surface of CMV infected inner ear cells, to mediate the protective effect. Findings from this study provide insight into the host immune response during CMV-induced hearing loss in mice.

## Introduction

Cytomegalovirus (CMV) is the most common infectious cause of congenital sensorineural hearing loss (SNHL) in humans [1] with between 15–30% of pediatric hearing loss attributable to this infection [2–4]. The consequences of hearing loss for affected children include speech and language delay, poor education attainment, and poor occupational performance in adulthood [5]. The total cost for each child with hearing loss is estimated to be over three hundred thousand dollars accounting for the lost productivity, the need for special education, vocational rehabilitation, assistive devices and medical costs [6]. One study estimates the total costs to the United States associated with congenital CMV infection to be $4 billion a year [7].

Despite the known significant health burden caused by congenital CMV induced hearing loss, very little is known about its pathogenesis including considerable uncertainty regarding the roles of direct viral replication in the cochlea and the contribution of host immune responses. An animal model that accurately recapitulates human CMV-induced hearing loss has been developed to evaluate more effective strategies for prevention and treatment [8, 9]. Our group and others have successfully demonstrated that murine CMV (mCMV)-induced labyrinthitis in BALB/c murine newborn pups occurs when green fluorescent protein (GFP) expressing mCMV was used to inoculate newborn mice via an intracerebral (IC) injection [10, 11]. These studies recapitulate viral mediated hearing loss in human infant because a critical factor for effective correlation between the mouse model and the clinical condition is that the mouse auditory system at birth is analogous to the human fetal auditory system and does not achieve stable thresholds until 4 weeks of age [12]. When infected at birth, fifty-five percent had profound hearing loss (≥ 80 dB) at 4 weeks of age, while the other forty-five percent initially showed moderate hearing loss that progressed to profound hearing loss by 6–8 weeks. These findings mirror the longitudinal human clinical studies that show that 50% of children with hearing loss have worsening thresholds over time [13, 14]. Moreover, asymmetric hearing loss occurred in 40% of the mice, similar to the rate of 50% among children with congenital CMV infection observed by Fowler and colleagues [15]. In addition, we also showed that this susceptibility to CMV-induced hearing loss was age dependent. While all of the postnatal day three (P3) infected mice showed elevated auditory brainstem response (ABR) thresholds at 4 weeks of age, only fifteen percent of the P8, and none of the P14 infected mice had hearing loss. Pass et al. demonstrated an age dependent effect in children with congenital CMV infection who were more likely to develop hearing loss when infected during the first trimester rather than later in pregnancy [16].

The mouse model is relevant to evaluate the viral host interactions that involve the innate and adaptive immune response [17, 18]. Previous studies have established that the response to mCMV infection is strain-dependent in mice, with susceptibility determined by expression of the Ly49H gene (Klra8 killer cell lectin-like receptor, subfamily A, member 8) in the distal portion of the natural killer cell gene complex [19–21]. During the early phase of infection, the Ly49H receptor recognizes m157, a viral-encoded class I major histocompatibility complex (MHC) homologue expressed at the cell surface of mCMV infected cells. Despite the adaptation of viral expression of the MHC as an immune evasion tactic [22], the interaction of m157 with the Ly49H receptor triggers NK cell activation and elimination of the infected cells in mice [23, 24]. Although Ly49H recognition of m157 is critical to controlling viral titers in the spleen and lung, it is much less effective in controlling virus replication in the liver. Here, we evaluated the role of NK cells, specifically Ly49H recognition of m157, in mCMV-induced labyrinthitis and subsequent hearing loss.

## Results

### C57BL/6 mice are protected from mCMV-induced hearing loss compared to BALB/c mice

**Fig 1:**
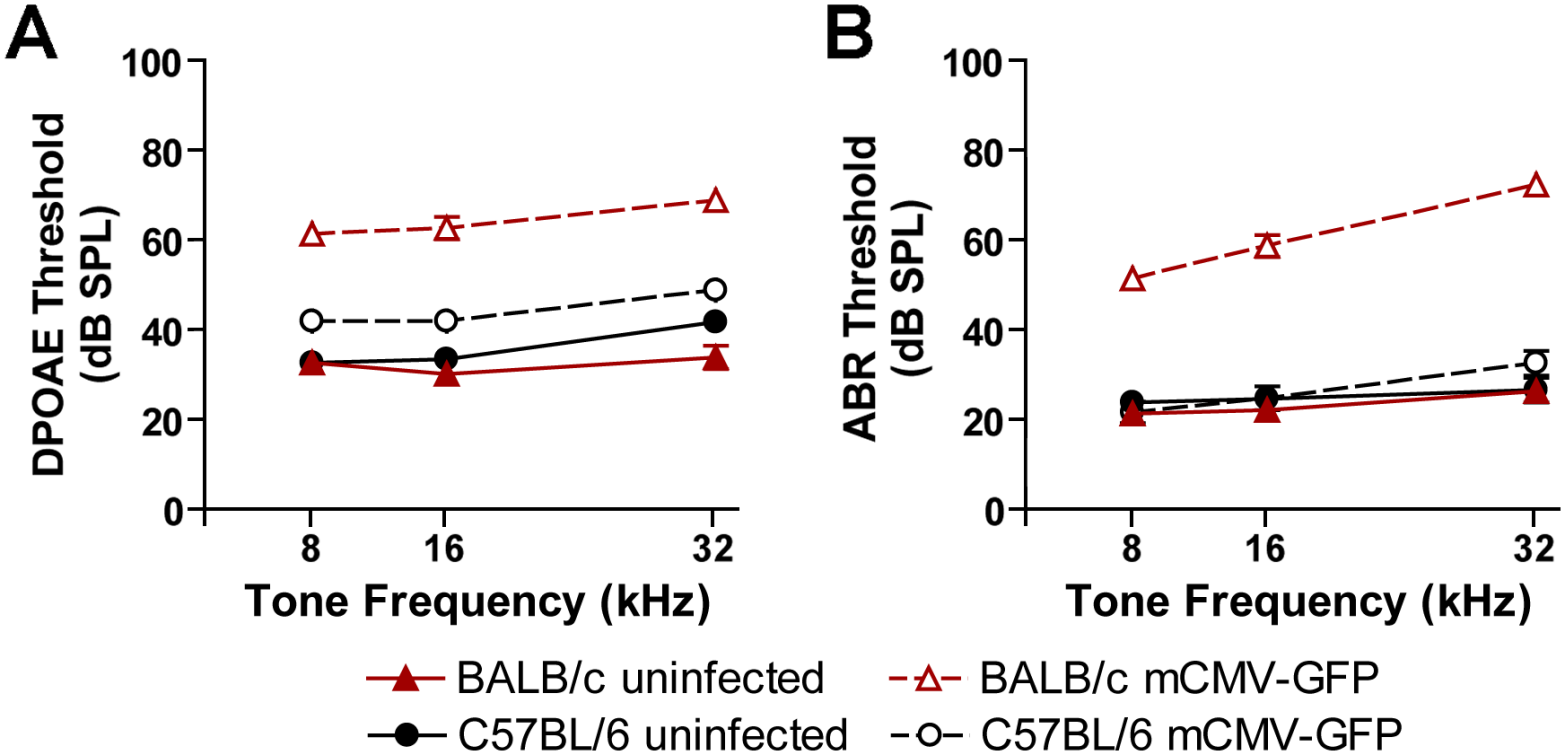
Hearing loss in mCMV-GFP infected BALB/c and C57BL/6 mice. Distortion product otoacoustic emission (DPOAE) (A) and auditory brainstem response (ABR) (B) thresholds of uninfected and infected mice were determined on post-natal day 28. Infected mice were treated with 200 pfu mCMV by intracerebral injection on post-natal day 3. Uninfected mice received the same volume of carrier by intracerebral injection on post-natal day 3. Statistically significant differences were seen between uninfected and infected BALB/c mice over the measured tone frequencies for both DPOAE and ABR thresholds (*P* < 0.0001, N = 6–7 mice per group) and between uninfected (N = 6) and infected (N = 12) C57BL/6 mice for DPOAE (*P* < 0.005), but not for ABR (*P* = 0.664). Statistically significant differences were also seen between infected BALB/c and C57BL/6 mice over the measured tone frequencies for both DPOAE and ABR thresholds (*P* < 0.0001). Statistical comparisons were performed using the Kruskal-Wallis test. ABR and DPOAE thresholds are presented as dB of sound pressure level (dB SPL) as a function of tone frequency in (kHz). Error bars represent standard error of the mean.

To analyze the physiological consequences of mCMV infection on hearing, we measured the minimum electrophysiological input required to evoke a threshold response of our experimental animals using auditory brainstem response (ABR), and distortion product otoacoustic emission (DPOAE). ABR samples evoked potentials in the auditory nerve and brainstem, thus measures the physiological response of the entire auditory pathway. DPOAE measures sound produced by the structures of the inner ear, in particular, the amplification function of the outer hair cells. In both cases, increased thresholds required to elicit measurable responses indicate hearing deficiency. ABR and DPOAE thresholds showed a marked difference between the two mouse strains. BALB/c pups inoculated at P3 with mCMV-GFP showed profound hearing loss by 4 weeks of age (Fig 1 and S1 Table). In contrast, infected C57BL/6 showed a slight, but significant, threshold elevation for DPOAE measurements compared to uninfected control mice (*P* < 0.005, Fig 1A and S1 Table) and no evidence of hearing loss based on ABR measurements (*P* = 0.664, Fig 1B and S1 Table). Comparison between infected C57BL/6 and BALB/c groups yielded a significant difference between the two strains over all DPOAE thresholds (*P* < 0.0001, S1 Table) and ABR thresholds (*P* < 0.0001, S1 Table). These data indicate that C57BL/6 mice were resistant to mCMV-induced hearing loss compared to BALB/c mice.

### C57BL/6 mice are protected from mCMV-induced outer hair cell loss compared to BALB/c mice

**Fig 2:**
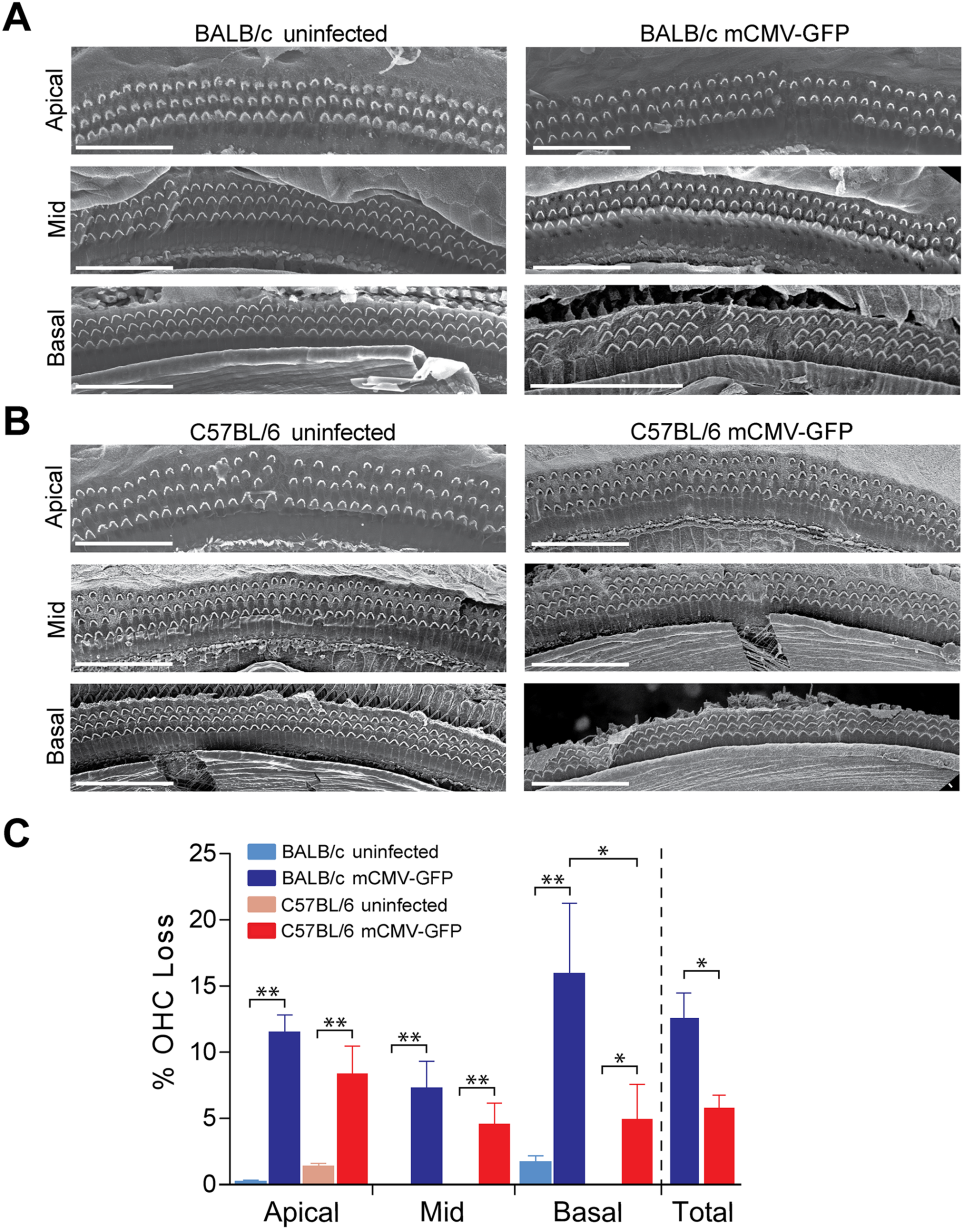
Outer hair cell loss in mCMV-GFP infected BALB/c and C57BL/6 mice. Representative scanning electron micrographs showing outer hair cells (OHC) from BALB/c (A) and C57BL/6 (B) mice infected at post-natal day 3 with mCMV-GFP (right panels) and uninfected controls (left panels). Scale bars represent 50 μm. (C) Quantification of outer hair cell loss from apical, mid, and basal turns is shown as well as for the total cochlea of infected mice at post-natal day 28. Data represents mean outer hair cell (OHC) loss from 4–5 mice per group. Error bars represents standard error of the mean. *P < 0.05 and **P < 0.001 by Mann–Whitney U test.

The outer hair cells (OHC) in the Organ of Corti function to enhance cochlear sensitivity and frequency selectivity and are responsible for amplification of sound vibrations measured by DPOAE. Since mCMV infection resulted in differential hearing loss, we evaluated outer hair cell loss in the two mouse strains. Outer hair cell loss was evident by scanning electron microscopy (SEM) in both BALB/c and C57BL/6 mice due to mCMV-GFP infection compared to uninfected controls (Fig 2A and 2B). However, total OHC loss was more than two-fold higher in infected BALB/c mice compared to C57BL/6 mice (Fig 2C). Compared to uninfected controls, OHC loss was evident in all cochlear turns, but BALB/c infected mice had more severe OHC loss in the basal cochlear turn than C57BL/6 infected mice (Fig 2C), perhaps reflecting the greater DPOAE and ABR thresholds seen at higher frequencies. Cochleograms reflected the differential OHC loss seen by SEM in the two strains (S1 Fig) and indicated greater loss of outer hair cells for BALB/c infected mice compared to C57BL/6 infected mice (S1B Fig). Although differences at individual time points did not reach the level of significance between the mouse strains, hair cell loss was progressive in that there was a significant overall time-dependent outer hair cell loss in BALB/c mice (*P* = 0.0053 by ANOVA). A similar time-dependent trend was noted for C57BL/6 mice that did not reach the level of significance (*P* = 0.109), but consistent with the resistance to mCMV hearing loss in C57BL/6 mice. These data indicate that strain-dependent hearing loss can be at least partially explained by OHC loss. Consistent with other forms of ototoxicity, mCMV infection did not markedly alter the appearance of inner hair cells when examined by SEM or immunohistochemistry and compared to uninfected controls (see images provided at https://doi.org/10.7278/S5V69GR6).

### C57BL/6 mice show increased and progressive hearing loss upon disruption of NK cell receptor/ligand interactions

**Fig 3:**
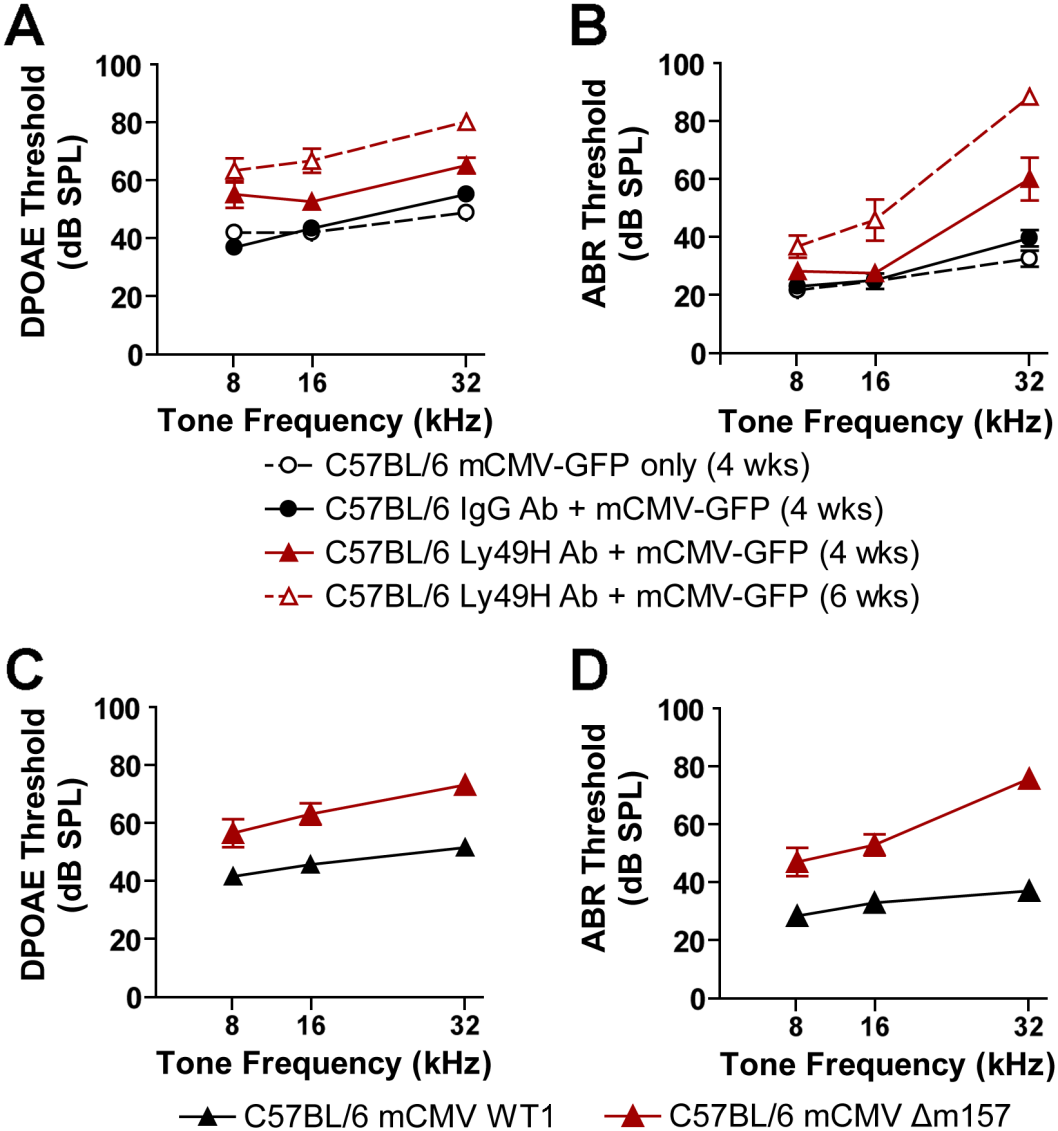
Hearing loss in mCMV infected C57BL/6 mice after disruption of NK cell recognition signals. The effect of Ly49H receptor blockade on hearing loss was evaluated by examining distortion product otoacoustic emission (DPOAE) (A) and auditory brainstem response (ABR) (B) thresholds in post-natal day 28 (4 wks) and post-natal day 42 (6 wks) after intraperitoneal injection of IgG isotype control antibody or Ly49H blocking antibody and/or 200 pfu mCMV-GFP delivered by intracerebral injection on post-natal day 3. Statistically significant threshold differences were seen between the infected mice treated with the IgG isotype control antibody (N = 12 mice) and infected mice treated with the Ly49H blocking antibody 4 weeks post-injection (N = 8 mice) for DPOAE (*P* < 0.0001) and ABR (*P* = 0.0001) over the measured tone frequencies. Infected C57BL/6 mice treated with anti-Ly49H antibody and mCMV-GFP showed significant progressive hearing loss at 6 weeks after inoculation compared to thresholds 4 weeks after inoculation (*P* = 0.001 for DPOAE, *P* < 0.0001 for ABR). The effect of the mCMV-encoded m157 immunoevasin ligand on hearing loss was tested by comparing DPOAE (C) and ABR (D) in C57BL/6 mice after infection with either a virus deleted for the m157 gene (mCMV Δm157) or its parental wild type virus (mCMV WT1). Statistically significant threshold differences (*P* < 0.001) were seen between the mCMV WT1 and mCMV Δm157 treated C57BL/6 groups (*P* < 0.0001 for both DPOAE and ABR). Statistical differences between groups were determined using the Kruskal-Wallis test. ABR and DPOAE thresholds are presented as dB of sound pressure level (dB SPL) as a function of tone frequency in (kHz). Error bars represent standard error of the mean.

To assess the direct participation of the NK cell Ly49H receptor/m157 ligand interaction in protecting C57BL/6 mice from mCMV-induced hearing loss, we first tested the effect of a Ly49H blocking antibody on DPOAE and ABR. Increased thresholds were seen at 4 weeks post-injection for all frequencies in mice receiving both the Ly49H blocking antibody and 200 pfu mCMV compared to mice receiving the IgG isotype control antibody and 200 pfu mCMV for DPOAE and ABR (*P* < 0.0001) (Fig 3A and S1 Table) although increases were most pronounced for ABR at the 32 kHz tone frequency (Fig 3B and S1 Table). This hearing loss worsened over time in that mCMV infected mice showed increases over all thresholds at 6 weeks compared to 4 weeks after inoculation (*P* < 0.0001). These data show that blocking the Ly49H receptor in otherwise resistant C57BL/6 mice resulted in at least mild hearing loss by 4 weeks of age that progressed to moderate-to-profound loss by 6 weeks after mCMV inoculation. We next tested the effect of the mCMV-encoded m157 ligand by inoculating C57BL/6 pups with a mCMV virus deleted for the m157 gene (mCMV Δm157, described in [25]). C57BL/6 mice infected with mCMV-Δm157 showed significant increases in both DPOAE (Fig 3C and S1 Table) and ABR (Fig 3D and S1 Table) thresholds over all tested frequencies relative to mice infected with wild-type mCMV virus. Taken together, these data indicate that a competent Ly49H receptor/m157 ligand interaction protects mice from mCMV-induced hearing loss.

### NK cells co-localize with mCMV-GFP infected cells in mouse cochlea

**Fig 4:**
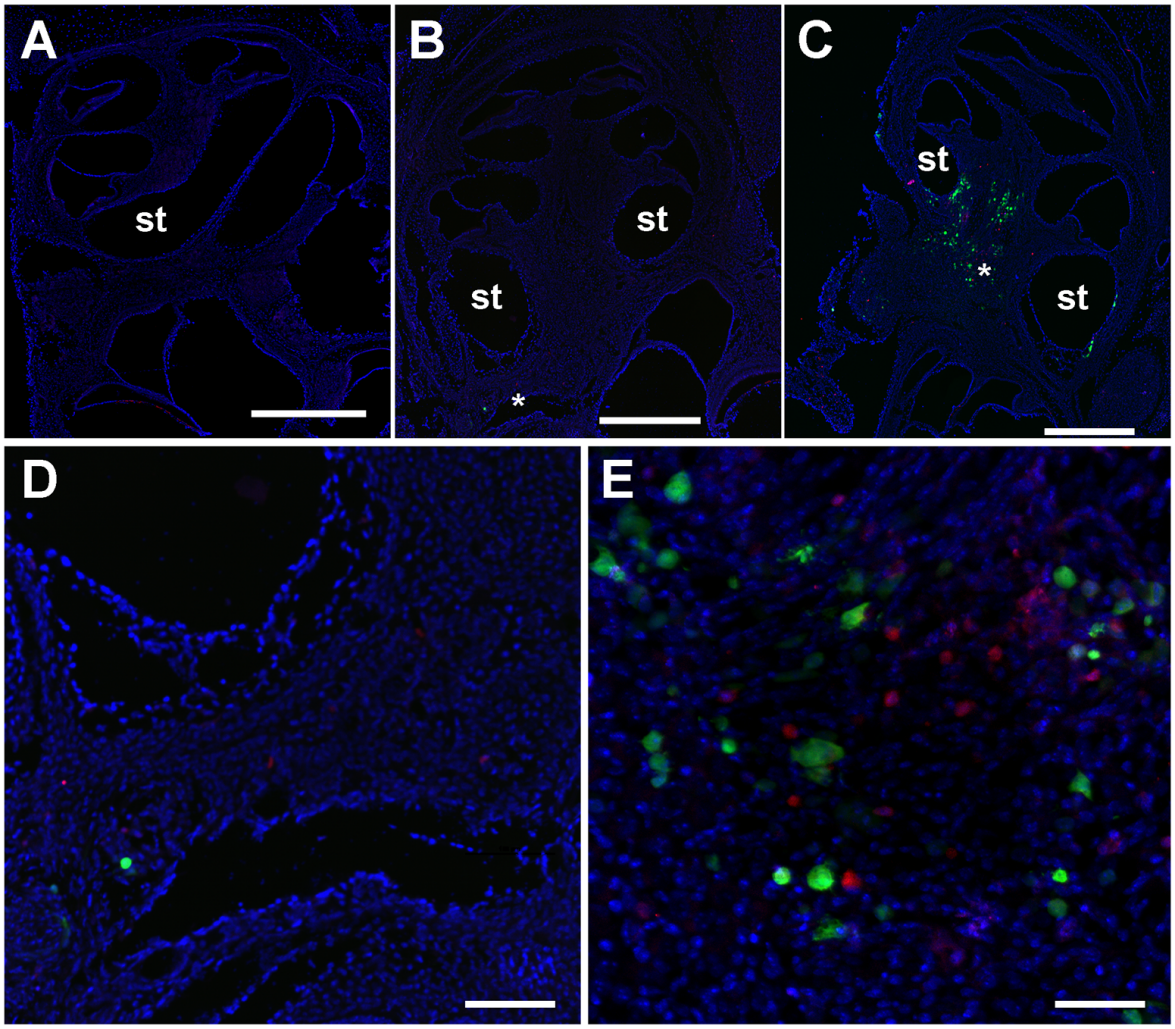
Ly49H blockade induces co-localization of mCMV-GFP infected cells and NK cells within the temporal bone. Green fluorescent protein expressed in mCMV-GFP infected cells and red fluorescent protein expressed in NK cells were visualized in cochlear cryosections from NK1.1-tdTomato knock-in mouse temporal bones harvested 3 days post-injection using anti-GFP (green) and anti-RFP antibodies (red). Representative images of 3–4 cochleae examined per group are shown for NK1.1-tdTomato knock-in mice injected with anti-Ly49H antibody only (A), mCMV-GFP only (B), or both (C). Panel D depicts a higher magnification of the auditory nerve region indicated by * in panel B. Panel E depicts a higher magnification of the spiral ganglion region indicated by * in panel C. Scale bars indicate 500 μm in panels A, B, and C and 100 μm in panels D and E. st = scala tympani.

In the absence of Ly49H/m157 disruption, mCMV infected cells in the C57BL/6 mouse cochlea were rare within the first week after infection, whereas mCMV infected cells were plentiful in cochlea from BALB/c mice up to one week post-infection [11]. Viral-encoded GFP was routinely detected in the spiral ganglion, and occasionally in perilymphatic epithelia by fluorescent and immunofluorescent microscopy (Fig 4 and S2 Fig) in C57BL/6 mice treated with Ly49H blocking antibody. Viral DNA was detected in temporal bones from infected mice (S3 Fig). Furthermore, activated caspase-3 was localized in the vicinity of GFP signals (S4 Fig) indicating mCMV infection resulted in activation of the apoptotic cascade. Caspase activation was only seen in coordination with GFP signal, which were largely confined to the spiral ganglion with rare individually infected cells seen in the scala tympani. Coordinated cleaved caspase signal dramatically increased within the spiral ganglion in mCMV infected C57BL/6 mice after Ly49H/m157 blockade. These data demonstrate active mCMV infection of cells within the mouse cochlea. However, consistent with our previous data in BALB/c mice [11], GFP signals, indicating active mCMV infection, were not seen in the hair cells of C57BL/6 mice at any of the examined time points up to 28 days post-infection suggesting that hair cells are not the direct target of mCMV. The effect of mCMV infection on NK cell localization was examined in a C57BL/6 mouse strain that constitutively expressed td-Tomato, a red fluorescent protein, in NK cells (NK1.1-tdTomato knock-in mice). NK cells, as indicated by RFP fluorescence (N = 6–12 mice per group) or RFP immunofluorescence (N = 3–4 mice per group), were rarely seen in in uninfected C57BL/6 mice and only occasionally seen in mCMV infected C57BL/6 mice (Fig 4B and 4D). In these cases, RFP signal appeared to be randomly localized. In contrast, pretreatment of NK1.1-tdTomato knock-in mice with Ly49H blocking antibody prior to mCMV-GFP inoculation showed a dramatic increase in both mCMV-GFP infected cells and associated NK cells three days after infection (Fig 4C and 4E). These data suggest that, in the presence of a competent Ly49H/m157 interaction, NK cells can effectively attenuate mCMV infection in the cochlea.

## Discussion

Natural killer cells are a fundamental component of the immune response to virally infected cells and are among the first immune cells to respond to pathogen challenge. NK cells function in the early innate response via cytolytic activity and affect the adaptive immune response through release of cytokines. In mice, recognition of virally infected cells is largely coordinated by the Ly49 C-type lectin family of homodimeric receptors, which have both activating and inhibitory isoforms. Numerous studies in mice have established that NK cell expression of the Ly49H receptor confers resistance to mCMV infection through early recognition of the virally-encoded m157 cell surface antigen [19, 21, 23, 24, 26, 27]. NK cell mediated protection from mCMV infection has been demonstrated by decreased viral load in murine spleen and lung [19, 25, 26, 28] and decreased cell lysis and tissue disintegration in spleen [26]. However, Ly49H/m157 interactions are less effective in other tissues such as the liver, so it was unclear if these interactions are relevant for mCMV-induced hearing loss in neonatal mice as we now demonstrate. DPOAE and ABR testing consistently showed elevated hearing thresholds and cochleograms demonstrated greater outer hair cell loss in the susceptible BALB/c mouse strain as compared to the near normal thresholds and minimal outer hair cell loss in the resistant C57BL/6 mouse strain. The fact that NK cell depletion by IP injection of neonatal mice (P2) with anti-asialo GM1 antibody increases mCMV titer and reduced cytokine production in brain indicates that the NK cell response participates in mCMV infection in newborn mice after IC injection [29]. Our data demonstrate that mCMV susceptibility to hearing loss is mediated, at least in part, by NK cells. We recognize that Ly49H receptor expression on NK cells has not been explicitly established and it remains possible that the blocking antibody interacts with an alternate target that results in hearing loss, however, that both addition of the Ly49H blocking antibody and infection with an m157 deficient viral strain rendered C57BL/6 mouse susceptible to mCMV hearing loss and cochlear damage suggests the Ly49H receptor/m157 interaction participates in mCMV-induced hearing loss. Additionally, NK cell co-localization with GFP expressing mCMV infected cells dramatically increased in the cochlea of normally resistant C57BL/6 mouse strain after blockade of the Ly49H receptor consistent with the requirement for physical interaction between NK cells and mCMV-infected cells for effective Ly49H engagement of m157.

Although a competent NK cell Ly49H receptor interaction with virally encoded m157 ligand in infected cells was required for otoprotection against viral infection in the cochlea, hearing was not completely preserved as DPOAE thresholds increased modestly, but significantly, in mCMV infected resistant C57BL/6 mice compared to uninfected controls. The increased DPOAE thresholds in resistant C57BL/6 mice were consistent with outer hair cell loss, which eventually reached about 50% of the loss seen in susceptible BALB/c mice, although overall OHC loss is minor and likely does not explain the full extent of hearing loss. These data indicate that a competent NK cell response is not sufficient to protect inner ear structures from damage after mCMV infection and that either NK cell clearance of infected cells was incomplete or that sequelae of infection contributed to subsequent SNHL. Furthermore, since direct evidence of mCMV infection in the hair or supporting cells within the organ of Corti was not seen suggests that secondary effects of mCMV infection are responsible for hair cell loss.

Our observation of outer hair cell loss without evidence of direct cochlear hair cell infection is consistent with previous studies of mCMV infected mice [10, 30, 31]. Similarly, mCMV infection favored cells of the spiral ganglion and perilymphatic epithelial cells, which is largely consistent with previous results [10, 30–32]. Apoptosis of spiral ganglion neurons has been identified as a component of hearing loss in the susceptible BALB/c mouse strain [32]. Our data demonstrates similar activation of the apoptotic cascade in the previously resistant C57BL/6 mouse strain after interruption of NK cell recognition signals suggesting that early clearance of mCMV infection by NK cells and protection from spiral ganglion apoptosis contributes to protection from hearing loss. The fact that spiral ganglion cells appear to be the major site of mCMV infection at 3-days post-infection after Ly49H receptor blockade indicates that protection of spiral ganglion cells was the main contributor to NK hearing loss protection.

It is known that individuals with defects affecting NK cell function are particularly susceptible to human CMV disease [33, 34]. Although the killer cell lectin-like receptor, subfamily A genes, of which Ly49H is a member, appear to be lacking in humans [35], human NK cells express a range of inhibitory and activating surface receptors, including lectin-like receptors and Ig-like receptors that could be explored in the context of CMV-induced hearing loss [36]. For example, a human CMV-encoded immunoevasin, UL18, has been shown to activate NK cells lacking leukocyte Ig-like receptor 1 [37]. However, it is unlikely that modulation of the NK cell response in the clinical setting will be a viable intervention target given the paucity of information about NK cell developmental status and receptor complement in utero or in newborns. Nevertheless, our results further delineate mechanisms of CMV-induced hearing loss in the mouse and provide additional evidence of the correlation to the clinical presentation of congenital CMV sensorineural hearing loss.

## Materials and methods

### Ethics statement

All animal studies were approved by the University of Utah Institutional Animal Care and Use Committee (protocol number 14–07006), performed in compliance with relevant institutional policies, local, state, and federal laws, and conducted following National Research Council Guide for the Care and Use of Laboratory Animals, Eighth Edition. Animals were anesthetized with hypothermia or ketamine/xylazine and euthanized by exsanguination after a surgical plane of anesthesia was reached.

### Viruses

Recombinant mCMV (strain K181 MC.55 (ie2- GFP+)) expressing green fluorescent protein (GFP) was supplied by Dr. Mark R Schleiss (Minneapolis, MN, USA). A mCMV mutant with a functional deletion of the m157 gene (Δm157) and its parental wild-type strain (WT1) were previously described [25, 38]. To grow viral stock, M2-10B4 murine fibroblast cells (cat# CRL-1972, American Type Culture Collection, Manassas, VA, USA) were cultured in complete medium (minimal essential medium, 10%(v/v) fetal bovine serum (FBS), 2mM l-glutamine, 100 U/ml penicillin, 0.1 mg/mL streptomycin, 10nM HEPES). Once 70% confluent, the viral inoculum was added and the cells were incubated to 80% to 100% cytopathic effect. Each plate was then subjected to three freeze–thaw cycles and the resulting viral supernatant was collected. Virus purification was carried out by a commercial contract laboratory (Virapur, San Diego, CA, USA) using the following protocol: cellular debris were removed by centrifugation (1,000×g) at 4°C, and the virus were pelleted through a 35% sucrose cushion (in Tris-buffered saline [50 mM Tris–HCl, 150 mM NaCl, pH 7.4]) at 23,000×g for 2 h at 4°C. The pellet was resuspended in Tris-buffered saline containing 10% FBS. Viral stock titers were determined on M2-104B cells as 50% tissue culture infective doses (TCID50) per milliliter.

### Mice

BALB/c, C57BL/6 mice (Jackson Labs, Sacramento, CA, USA) and a C57BL/6 mouse strain that constitutively expresses red fluorescent protein (NK1.1-tdTomato knock-in mice) in NK and NKT cells [39] were used for experiments as indicated. Animals were housed and bred under pathogen-free conditions at the Central Animal Facility at the University of Utah.

Mice were injected via an intracerebral route at post-natal day 3 (P3) of life as previously described [11]. Briefly, the pups were momentarily placed on ice to induce anesthesia. The mouse was manually restrained, and a 10 μl Hamilton syringe with a 30G needle was inserted past the calvarium in the mid parietal region to inject 200 plaque forming units (pfu) of virus in a volume of 1 μl. Control groups received 1 μl phosphate-buffered saline (PBS) carrier unless otherwise indicated. Mice were monitored for adverse effects, including mortality, behavioral abnormalities, and developmental delay. Experimental and control animals were housed separately. For groups pretreated with Ly49H blocking antibody, 20 μg of purified mouse anti-Ly49H monoclonal antibody (clone 3D10, cat# 14-5886-82, eBiosciences, San Diego, CA, USA) in 50 μl of PBS was injected into the peritoneal cavity twelve hours before the inoculation of mCMV. Control mice received 20 μg of purified mouse IgG isotype control antibody (cat# 02–6502, ThermoFisher, Rockford, IL, USA) in 50 μl of PBS.

### Hearing assessment

For auditory brainstem response (ABR) and distortion product otoacoustic emission (DPOAE) testing, mice were anesthetized with a combination of ketamine and xylazine at 100 and 10 mg/kg body weight, respectively. ABRs/DPOAEs were performed in a double-walled sound chamber (IAC Acoustics, North Aurora, IL, USA). The body temperature was maintained at ~37°C via a heating pad. A small incision was made at the tragus to allow better access to the ear canal. For ABR testing, an electrostatic speaker (EC-1, Tucker-Davis Technology, Alachua, FL, USA) fitted with a 1.5 cm long polyethylene tube was placed abutting the ear canal. Needle electrodes were placed subcutaneously at the mastoid of the tested side and vertex, with a remote ground electrode placed in the rump area. ABR thresholds were measured bilaterally in all mice. ABR signals were amplified with a TDT RA4 pre-amplifier (Tucker-Davis Technology), filtered from 100 to 3000 Hz, averaged and digitized with a TDT RA16BA processor controlled by BioSigRP software (Tucker-Davis Technology). Acoustic stimuli were digitally generated and processed by a RX6 real-time processor and passed through a PA5 attenuator prior to delivery to the speaker amplifier at a rate 24–32 times/sec. Responses to 1,000 sweeps were averaged for a series of responses to tone pips ranging from 8 to 32 kHz (5 ms with 0.5 ms cos2 rise and fall) using 5 or 10 dB intensity steps, over a 15–90 dB of sound pressure level (dB SPL) range. ABR traces were visually inspected after plotting the amplitude of each peak against stimulus intensity. Thresholds typically corresponded to a level one step below that at which the peak-to-peak response amplitude began to rise. An ABR threshold of 90 dB SPL (i.e., the highest stimuli presented in this study) was assigned to cochleae that failed to stimulate an ABR waveform at 90 dB SPL. The DPOAEs were measured using an ER-10B+ (Etymotic Research, Elk Grove, IL, USA) microphone coupled with two EC1 speakers. Stimuli of two primary tones f1 and f2 (f2/f1 = 1.2) were presented with f2 = f1–10 dB. Primary tones were stepped from 30 to 80 dB SPL (for f1) in 10 dB increments and swept from 8 to 32 kHz in octave steps. Stimuli were generated and attenuated digitally (200 kHz sampling). The ear canal sound pressure was pre-amplified and digitized. A fast Fourier transformation was computed, and the sound pressures at f1, f2, and 2f1- f2 were extracted after spectral averaging from 50 serial waveform traces (each corresponding to 84 ms of digitized ear canal sound pressure waveform). The noise floor (average of 10 points in the FFT on either side of 2f1-f2) was also measured: it ranged between -25 and 0 dB SPL, depending on the test frequencies. All data were shown in mean ± SEM. The mice hearing reaches mature thresholds by 3 weeks of age [12]. To avoid potential confounding results due to immaturity of the auditory system in mCMV infected mice, we chose to test our animals beginning 4 weeks of age.

### Immunofluorescent histology

Mice were anesthetized with ketamine/xylazine and exsanguinated by transcardial (left ventricle to right atrium) perfusion with 0.1 M phosphate-buffered saline (PBS), pH 7.4 containing 100 U/ml heparin followed by 20 ml of 2% paraformaldehyde in phosphate buffer (PB) at room temperature (RT). The excised cochleae were immersed in fixative (2% paraformaldehyde in 0.1 M PB) overnight at 4°C, washed with PBS, and decalcified by immersion in 0.12 M EDTA, pH 7.0 for 1–5 days at 4°C. The decalcified cochleae were infiltrated with sucrose and then embedded in 7.5% gelatin/15% sucrose/1 X PBS. Serial 10 μm thick mid-modiolar sections were cut on a freezing microtome and mounted on poly-L-lysine-coated glass slides. Slides were stored at −20°C until further use. The slides were dried at RT for 10 min, washed in PBS, permeabilized with 0.2% Triton X-100 in PBS for 1 hour at RT, washed in PBS, and transferred to blocking buffer (BlockAid Blocking Solution, ThermoFisher, Waltham, MA, USA) for 1 hour at RT, prior to application of primary antibodies.

Primary antibodies to GFP (goat polyclonal anti-GFP, cat# AF4240, R&D Systems, Minneapolis, MN, USA) and tdTomato (rabbit polyclonal anti- RFP, cat#600-401-379, Rockland Antibodies, Limerick, PA, USA) were also used to validate the fluorescent labels. Rabbit anti- Myosin VIIa (polyclonal, cat# 25–6790, Proteus BioSciences, Ramona, CA, USA) was used to visualize hair cells in whole-mount cochleograms. Rabbit anti- active caspase-3 (clone C92-605, cat# 559565, BD Pharmingen, San Jose, CA, USA) was used to monitor apoptosis. All antibodies were incubated in blocking buffer. Following overnight incubation of the sections in primary antibodies at 4°C, the sections were rinsed in PBS and secondary antibodies applied for 1 hour at RT. Secondary antibodies used were donkey anti-goat IgG, Alexa Fluor 488 conjugate (cat# A11055, ThermoFisher) for the GFP primary, chicken anti-rabbit IgG, Alexa Fluor 594 conjugate (cat# A21442, ThermoFisher) for the RFP and Myosin VIIa primary antibodies, and donkey anti-rabbit IgG, Alexa Fluor 568 conjugate (cat# A10042, ThermoFisher) for the caspase-3 primary. Sections were counterstained with 4’,6-diamidino-2-phenylindole (DAPI) in PBS for 5 min at RT in the dark.

### Scanning electron microscopy

Scanning electron microscopy (SEM) was performed as described previously [40]. Briefly, mice were anaesthetized and then exsanguinated using 2.5% glutaraldehyde in PBS via transcardial perfusion. Temporal bones were harvested and fixed in 2.5% glutaraldehyde/PBS overnight at 4°C. Cochleas were dissected and coated using 1% osmium tetroxide and thiocarbohydrazide, followed by critical point drying using hexamethyldisilazane. Samples were then imaged using Hitachi-4800 scanning electron microscope.

### Cochleograms

Mice and temporal bones were treated and processed as described for histochemistry in the main text, through the decalcification step. Mouse cochlear whole mounts were prepared as described in (http://www.bio-protocol.org/e332). Hair cells were visualized using a primary antibody to myosin VIIA (rabbit anti-myosin VIIA, Proteus Biosciences cat# 25–6790, Ramona, California) and chicken anti-rabbit Alexa Fluor 594 (Life Technologies cat# A-21442) secondary antibody. A standard cochleogram was prepared for each ear using a 20X objective on a Nikon A1R confocal microscope. In each section, the number of present and absent hair cells was assessed throughout the entire section thickness and plotted as fractional loss.

### Viral DNA analysis

Viral DNA was measured by quantitative PCR (qPCR) using the viral Immediate Early response gene 1 (IE1) as the target relative to β-actin expression. DNA was extracted from the crushed temporal bones using QIAmp MinElute Virus Spin Kit (#57704, Qiagen, Valencia, CA). Each sample was assayed in duplicate using Taqman Gene Expression Master Mix (# 4370048, Life Technologies, Carlsbad, CA) and the Applied Biosystems QuantStudio 12K Flex Real Time PCR System (Life Technologies). Amplification conditions were initial denaturation for 95°C for 10 minutes, followed by 45 cycles of denaturation at 95°C for 15 seconds and anneal/extension at 60°C for 1 minute. Primer sequences were: IE1 primer 1: CCC TCT CCT AAC TCT CCC TTT, IE1 primer 2: TGG TGC TCT TTT CCC GTG, ActinB primer 1: AGC TCA TTG TAG AAG GTG TGG, Actin B primer 2: GGTGGG AAT GGG TCA GAAG (Integrated DNA Technologies, Prime Time Standard qPCR Assay 6FAM/Zen/ABFQ). Delta Ct values were determined for IE1 DNA levels normalized relative to β-actin levels and comparisons between groups were carried out using nonparametric rank sums tests. A standard curve was generated using DNA extracted from known quantities of mCMV. Efficiency of the PCR reaction was 99.66% as determined from the slope of the CT vs. log(mCMV DNA) standard curve.

### Statistical analyses

Statistical analysis was carried out in IBM SPSS Statistics for Windows (V. 21, IBM, Armonk, NY, USA) and GraphPad Prism for Windows (V. 6, GraphPad Software, La Jolla, CA, USA). Data are expressed as mean ± SEM. Differences in ABR and DPOAE thresholds were analyzed by means of the nonparametric Kruskal-Wallis test. OHCs loss from cochleas imaged using SEM was analyzed by means of Mann–Whitney U test. Time-dependent cochleogram data were analyzed by means of 2-way ANOVA. The *a priori* significance level was set at *P* < 0.05.

## Supporting information

S1 Fig mCMV-GFP infection results in outer hair cell loss. (PDF)

S2 Fig mCMV-GFP infection results in NK cell recruitment in mouse cochlea. (PDF)

S3 Fig Viral DNA can be detected in mouse cochlea after mCMV-GFP infection. (PDF)

S4 Fig mCMV-GFP infected cells activate apoptotic cascade. (PDF)

S1 Table DPOAE and ABR statistical comparisons between groups. (PDF)
