# Supplementary material for: Natural killer cells attenuate cytomegalovirus-induced hearing loss in mice
Source: PLoS Pathog. 2017 Aug 31;13(8):e1006599. doi: 10.1371/journal.ppat.1006599 (PMC5597263; doi:10.1371/journal.ppat.1006599)
Supplement: S1 Fig — (PDF) [file ppat.1006599.s001.pdf]

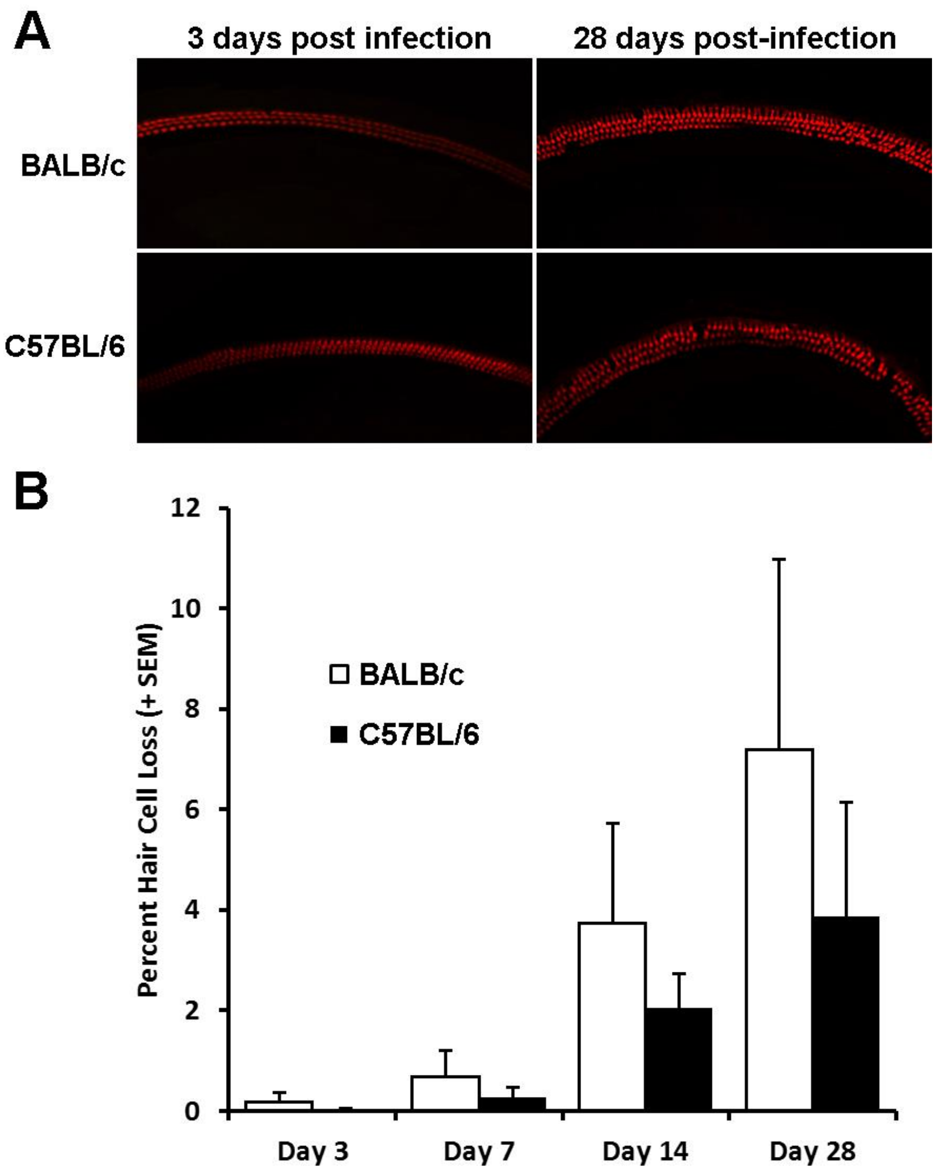

S1 Fig. mCMV-GFP infection results in outer hair cell loss.

(A) Representative cochleograms showing outer hair cells for BALB/c (top panels) and C57BL/6 (bottom panels) mice harvested 3 (left panels) and 28 (right panels) days after. (B) Quantitation of outer hair cell loss across the length of the cochlear partition at 3, 7, 14 and 28 days after inoculation showed less hair cell loss at each time point in C57BL/6 mice relative to BALB/c mice, although differences at individual time points did not reach the level of significance between the mouse strains (Mann-Whitney U test, N = 3-5 mice/group). However, there was a significant overall time-dependent outer hair cell loss in BALB/c mice (P = 0.0053 by ANOVA). A similar comparison for C57BL/6 mice did not reach the level of significance (P = 0.109). Error bars represent standard error of the mean (SEM).
