# Supplementary material for: Natural killer cells attenuate cytomegalovirus-induced hearing loss in mice
Source: PLoS Pathog. 2017 Aug 31;13(8):e1006599. doi: 10.1371/journal.ppat.1006599 (PMC5597263; doi:10.1371/journal.ppat.1006599)
Supplement: S2 Fig — (PDF) [file ppat.1006599.s002.pdf]

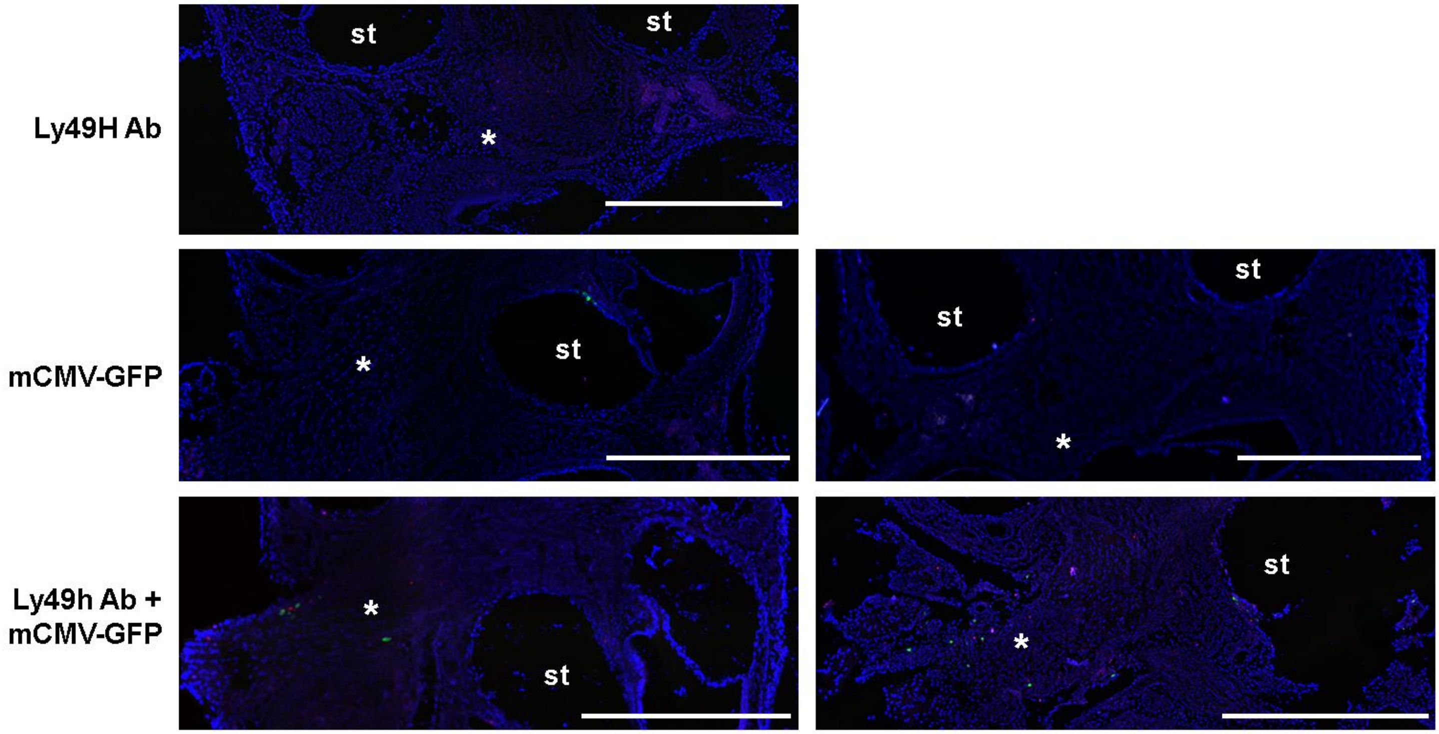

S2 Fig. mCMV-GFP infection results in NK cell recruitment in mouse cochlea.

Green fluorescent protein expressed in mCMV-GFP infected cells and red fluorescent protein expressed in NK cells were visualized in cochlear cryosections from NK1.1-tdTomato knock-in mouse temporal bones harvested 3 days post-injection using anti-GFP (green) and anti-RFP antibodies (red). Images are shown for additional NK1.1-tdTomato knock-in mice injected with anti-Ly49H antibody only, mCMV-GFP only, or both anti-Ly49H antibody and mCMV-GFP. Asterisks indicate comparable area of the spiral ganglion region. Scale bars represent 500 µm. st = scala tympani
