# Supplementary material for: Natural killer cells attenuate cytomegalovirus-induced hearing loss in mice
Source: PLoS Pathog. 2017 Aug 31;13(8):e1006599. doi: 10.1371/journal.ppat.1006599 (PMC5597263; doi:10.1371/journal.ppat.1006599)
Supplement: S3 Fig — (PDF) [file ppat.1006599.s003.pdf]

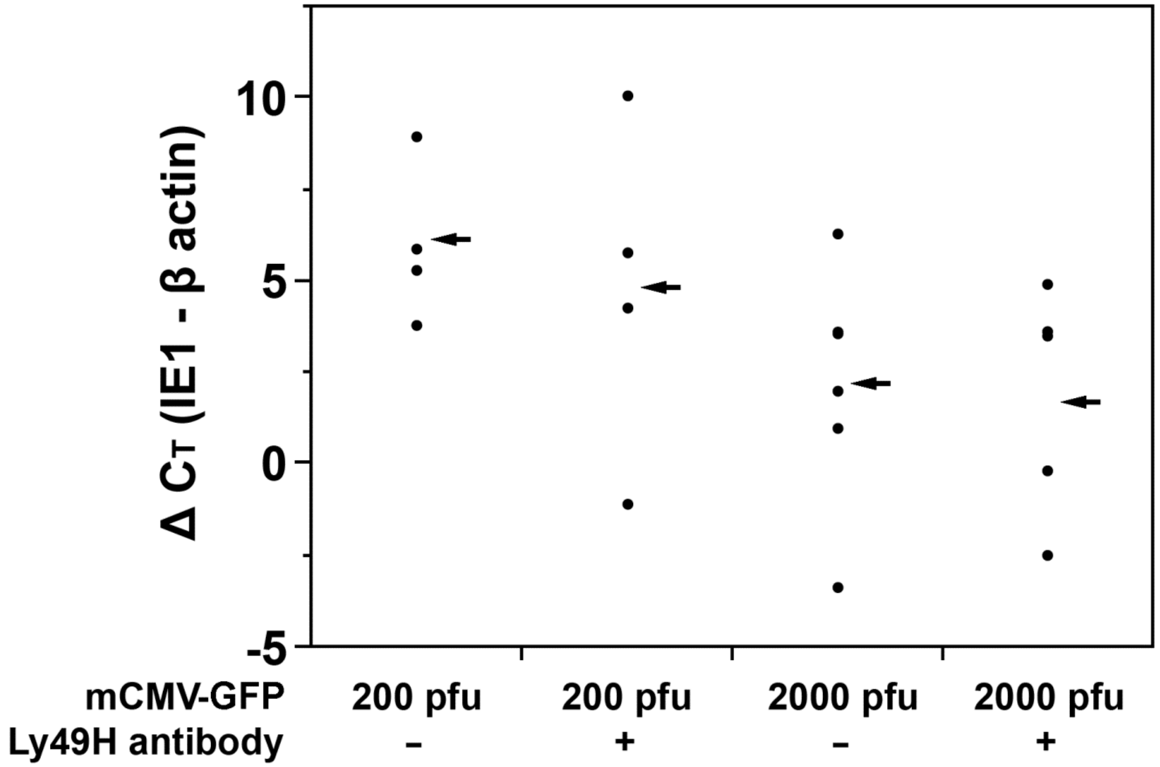

S3 Fig. Viral DNA can be detected in mouse cochlea after mCMV-GFP infection.

Quantitative PCR was performed using DNA extracted from C57BL/6 mouse temporal bones 3 days after intra-cerebral injection with mCMV-GFP, with and without prior injection with Ly49H blocking antibody. Normalized levels of mCMV IE1 DNA were determined by the delta CT method. Mean values in each group are indicated by arrows. DNA concentration is inversely related to delta CT with a smaller delta CT value indicating higher target DNA signal. Comparisons between groups did not reach the level of significance (*P* = 0.124 by Wilcoxon/Kruskal-Wallis rank sums test) although a trend towards increased IE1 DNA was evident by the smaller delta CT values after injection with 2000 pfu mCMV-GFP as compared to 200 pfu mCMV-GFP.
