# Supplementary material for: Natural killer cells attenuate cytomegalovirus-induced hearing loss in mice
Source: PLoS Pathog. 2017 Aug 31;13(8):e1006599. doi: 10.1371/journal.ppat.1006599 (PMC5597263; doi:10.1371/journal.ppat.1006599)
Supplement: S4 Fig — (PDF) [file ppat.1006599.s004.pdf]

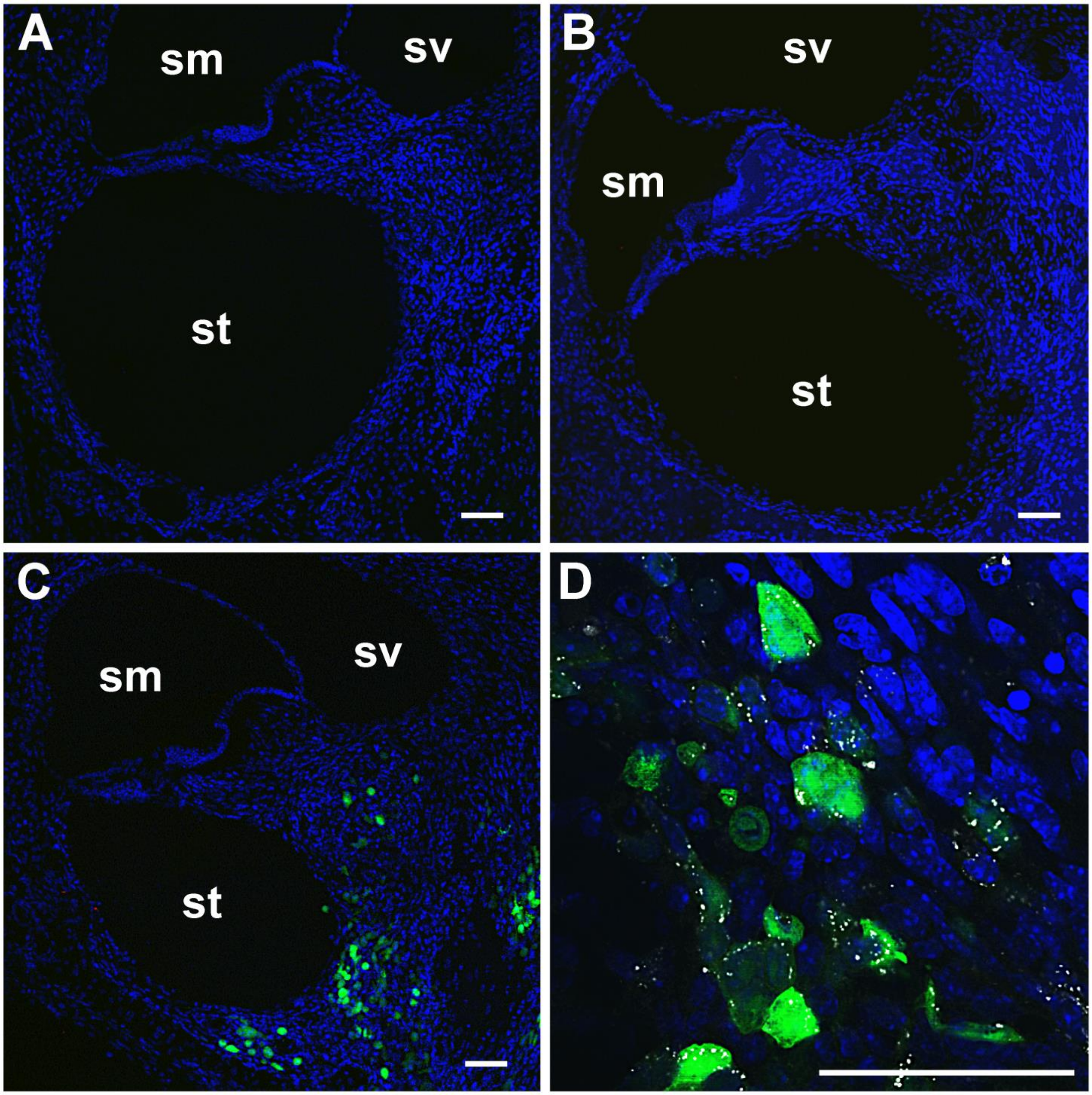

S4 Fig. mCMV-GFP infected cells activate apoptotic cascade.

Cochlear cryosections from mice treated with Ly49H blocking antibody only (A), 200 pfu mCMV-GFP inoculation at P3 only (B) or both Ly49H blocking antibody and mCMV-GFP (C) were evaluated 3 days post-injection using anti-cleaved caspase-3 antibodies (white). Cleaved caspase signal appears co-localized with cells expressing native GFP signal, indicating mCMV infection (green). Panel D depicts a higher magnification of the spiral ganglion from the Ly49H+mCMV-GFP treated group. Representative images of 3-4 cochleae per group are shown. Scale bars indicate 50 µm. st = scala tympani, sm = scala media, sv = scala vestibuli
