# Supplementary material for: Natural killer cells attenuate cytomegalovirus-induced hearing loss in mice
Source: PLoS Pathog. 2017 Aug 31;13(8):e1006599. doi: 10.1371/journal.ppat.1006599 (PMC5597263; doi:10.1371/journal.ppat.1006599)
Supplement: S1 Table — (PDF) [file ppat.1006599.s005.pdf]

S1 Table. DPOAE and ABR statistical comparisons between groups.
Comparisons were modeled over all frequencies by the Kruskal-Wallis test.

Strain	Treatment	N	DPOAE Model (*P*-values)	ABR Model (*P*-values)
C57BL/6	uninfected (IC carrier)	6		
C57BL/6	mCMV-GFP	12	<0.005	0.664
BALB/c	uninfected (IC carrier)	7		
BALB/c	mCMV-GFP	6	<0.0001	<0.0001
C57BL/6	mCMV-GFP	12		
BALB/c	mCMV-GFP	6	<0.0001	<0.0001
C57BL/6	IgG Ab + mCMV-GFP (4 wks)	12		
C57BL/6	mCMV-GFP (4 wks)	12	0.230	0.782
C57BL/6	IgG Ab + mCMV-GFP (4 wks)	12		
C57BL/6	Ly49H Ab + mCMV-GFP (4 wks)	8	<0.0001	<0.0001
C57BL/6	Ly49H Ab + mCMV-GFP (4 wks)	8		
C57BL/6	Ly49H Ab + mCMV-GFP (6 wks)	6	<0.0001	<0.0001
C57BL/6	mCMV WT1	6		
C57BL/6	mCMV Δm157	12	<0.0001	<0.0001
